# Activation of hypoxia signaling induces phenotypic transformation of glioma cells: implications for bevacizumab antiangiogenic therapy

**DOI:** 10.18632/oncotarget.3592

**Published:** 2015-03-14

**Authors:** Hui Xu, Shervin Rahimpour, Cody L. Nesvick, Xu Zhang, Jingyun Ma, Min Zhang, Ge Zhang, Li Wang, Chunzhang Yang, Christopher S. Hong, Anand V. Germanwala, J. Bradley Elder, Abhik Ray-Chaudhury, Yu Yao, Mark R. Gilbert, Russell R. Lonser, John D. Heiss, Roscoe O. Brady, Ying Mao, Jianhua Qin, Zhengping Zhuang

**Affiliations:** ^1^ Division of Biotechnology, Dalian Institute of Chemical Physics, Chinese Academy of Sciences, Dalian, China; ^2^ Surgical Neurology Branch, National Institute of Neurological Disorders and Stroke, Bethesda, Maryland, USA; ^3^ Department of Immunology, Dalian Medical University, Dalian, China; ^4^ Department of Neurological Surgery, Loyola University Medical Center, Chicago, Illinois, USA; ^5^ Department of Neurological Surgery, The Ohio State University Medical Center, Columbus, Ohio, USA; ^6^ Department of Neurosurgery, Huashan Hospital, Shanghai Medical College, Fudan University, Shanghai, China; ^7^ Department of Neuro-Oncology, Division of Cancer Medicine, The University of Texas MD Anderson Cancer Center, Houston, Texas, USA

**Keywords:** glioblastoma, bevacizumab, epithelial-mesenchymal transition, pathologic angiogenesis, hypoxia-inducible factor

## Abstract

Glioblastoma (GBM) is the most common and deadly primary brain tumor in adults. Bevacizumab, a humanized monoclonal antibody against vascular endothelial growth factor (VEGF), can attenuate tumor-associated edema and improve patient symptoms but based on magnetic resonance imaging, is associated with non-enhancing tumor progression and possibly gliosarcoma differentiation. To gain insight into these findings, we investigated the role of hypoxia and epithelial-mesenchymal transition (EMT)-associated proteins in GBM. Tumor markers of hypoxia and EMT were upregulated in bevacizumab-treated tumors from GBM patients compared to untreated counterparts. Exposure of glioma cells to 1% oxygen tension increased cell proliferation, expression of EMT-associated proteins and enhanced cell migration *in vitro*. These phenotypic changes were significantly attenuated by pharmacologic knockdown of hypoxia-inducible Factor 1α (HIF1α) or HIF2α, indicating that HIFs represent a therapeutic target for mesenchymal GBM cells. These findings provide insights into potential development of novel therapeutic targeting of angiogenesis-specific pathways in GBM.

## INTRODUCTION

Glioblastoma (GBM) is the most common adult primary nervous system tumor. Despite advances in surgical resection, radiation and chemotherapy, GBM remains one of the most deadly human neoplasms. GBM patients have a median survival of 12 to 15 months and new therapies are desperately needed [1]. Bevacizumab, a humanized monoclonal antibody against vascular endothelial growth factor (VEGF), has been shown to improve progression-free survival in patients with recurrent glioblastoma [2-4]. As one of the most highly vascular cancers, GBMs express high levels of VEGF, particularly in areas of necrosis and hypoxia [5, 6]. The increased levels of VEGF expression and vascular density in GBM make angiogenesis an attractive therapeutic target. Clinical trials have demonstrated that bevacizumab is a therapeutic option for recurrent GBM patients who have failed previous radiation and chemotherapy [3, 7].

Angiogenesis inhibitors, including bevacizumab, produce demonstrable transient clinical and radiological benefits for patients with a variety of cancer types including GBM [8]. However, in 40 to 60% of cases, initial responses are frequently followed by dramatic progression of disease [2, 9]. Consequently, overall survival has not been significantly improved with anti-angiogenic therapy and is associated with an increased rate of transformation to secondary gliosarcoma [2-4, 9, 10]. Recent data indicate that resistance to bevacizumab anti-angiogenic therapy can be due to evasive (upregulation of alternative pro-angiogenic pathways) or intrinsic (genomic constitution) changes within the neoplasm [11]. These findings potentially make combinatorial strategies, specifically integration of both anti-angiogenic therapy and anti-resistance mechanisms, particularly attractive for managing GBM.

Critical to a deeper understanding of the pathobiology of therapeutic resistance and progression will be insights into the effects of anti-angiogenic therapy in GBM. To better understand the mechanisms that underlie tumor cell invasiveness and progression of disease during/following anti-angiogenic therapy, we examined the phenotypic changes of GBM cells in the setting of induced hypoxia. Specifically, bevacizumab-induced inhibition of VEGF can trigger intratumoral hypoxia and initiate compensatory survival pathways, namely upregulation of hypoxia-inducible factors (HIFs) [12]. Data indicate that HIF stabilization enhances tumor cell invasion, cell growth and cell survival and thus serves a critical role in modulating tumor aggression [13-22]. This may underlie the clinical and radiographic findings associated with anti-angiogenic therapy in GBM patients.

Based on the emerging clinical and imaging findings in recurrent GBM patients treated with bevacizumab, we hypothesized that the lack of improved overall survival in these patients is modulated through the activation of HIF-mediated survival pathways. To test this hypothesis, we analyzed expression levels of HIF down-stream effectors and epithelial-to-mesenchymal (EMT) markers as well as microfluidic invasion assays of GBM cells under normoxic and hypoxic conditions. Moreover, glioma cell phenotype and migration were analyzed following HIF inhibition and gain-of-function to investigate the role of HIFs in tumor cell aggressiveness/progression. Finally, these findings were correlated with comprehensive immunohistochemical (IHC) analysis of recurrent GBM patients treated with bevacizumab via comparative analysis of tumor tissue before and after treatment.

## RESULTS

### Hypoxia and mesenchymal transition in human GBM after anti-angiogenic therapy

**Figure 1:**
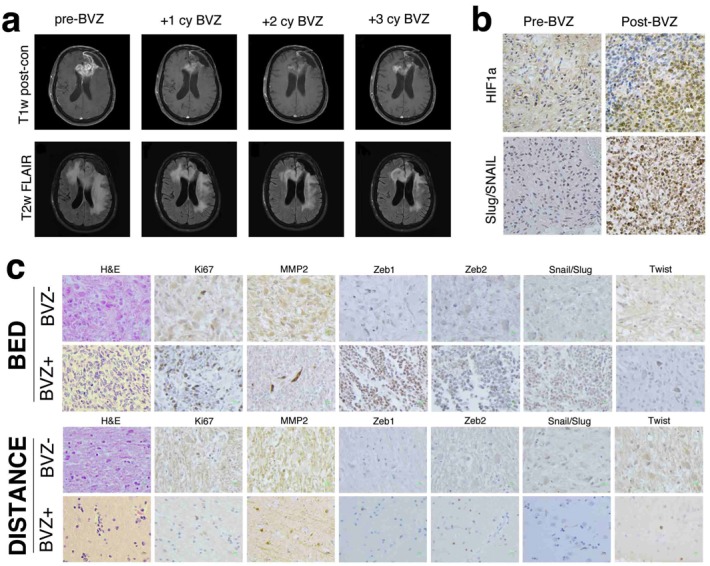
MR imaging and immunhistochemistry of glioblastoma before and after bevacizumab therapy (a) MR findings in glioblastoma before and after bevacizumab therapy. T1 post-contrast and fluid-attenuated inversion recovery (FLAIR) axial MR images before, after one cycle, two cycles and three cycles or bevacizumab therapy. An initial reduction in the enhancing portions of the residual tumor involving the left frontal lobe and corpus callosum is seen, and a decrease in overall cerebral edema is observed. However, these radiographic improvements are temporary, and later imaging demonstrates increased enhancement and edema. (b) Immunohistochemical staining of HIf1α and the EMT inducers Slug and Snail in a glioblastoma surgical specimen before (left) and after (right) bevacizumab therapy. Following antiangiogenic therapy, an increase in HIF1α expression is seen concomitant with increased expression of Slug and Snail. Magnification 400 ×. (c) Hematoxylin and eosin (H&E) and immunohistochemical stains of multiple EMT inducers in glioblastomas treated with temozolomide and radiation therapy with and without adjuvant bevacizumab. Compared to the tumor treated with radiation and temozolomide, the bed of the bevacizumab-treated tumor exhibits markedly increased cellularity, Ki67-positivity and a significant increase in the number of spindle-shaped mesenchymal cells. The tumor bed also demonstrated an increase in the number of cells positive for the EMT inducers Zeb1, Zeb2, Slug, Snail, Twist and the EMT marker matrix metalloproteinase 2 (MMP2). Stains performed on sections five centimeters from the tumor bed revealed periodic single infiltrating cells that were often positive for each of the EMT inducers, but no discernible difference was seen between the tumors. Magnification 400 ×.

Bevacizumab treatment of recurrent GBM is commonly associated with a decrease in intratumoral enhancement and peri-tumoral edema. The reduction in edema results in alleviation of tumor-associated symptoms (Fig. 1a). However, these effects are transient and the tumor eventually becomes refractory to therapy, demonstrates increased infiltration of surrounding brain. and is associated with transformation to gliosarcoma [10]. To test the hypothesis that anti-angiogenic therapy can induce an EMT-like process through hypoxia in GBM, we analyzed tumor tissues from three recurrent GBM patients for markers of hypoxia and EMT before and after bevacizumab treatment. Tumor histology from Patient 1 was most consistent with GBM before bevacizumab therapy but showed histologic changes consistent with transformation to gliosarcoma after treatment (Fig. 1b). Tumor tissues revealed markedly elevated expression of HIF1α and EMT markers Slug and Snail (diffuse pattern), suggesting that the hypoxic microenvironment activated an EMT-like process post-bevacizumab therapy.

Brains from Patients 2 and 3 were examined postmortem. While both patients received radiation and temozolomide chemotherapy, Patient 3 also received bevacizumab (Fig. 1c). Compared to the tumor from Patient 2, the bevacizumab-treated tumor (Patient 3) exhibited a marked increase in cellularity, cell proliferation and spindle-shaped mesenchymal morphology. Furthermore, the bevacizumab-treated tumor contained significantly more tumor cells that stained for the EMT markers matrix metalloproteinase 2 (MMP2), Zinc-finger E box-binding homeobox 1 (Zeb1), Zeb2, Snail, Slug and Twist. Sections taken 5 cm away from the tumor mass revealed occasional single infiltrating tumor cells positive for Ki67 and each of the EMT markers, but the scarce number of infiltrating cells precluded any between-tumor comparisons.

### Hypoxia enhances GBM cell proliferation

**Figure 2:**
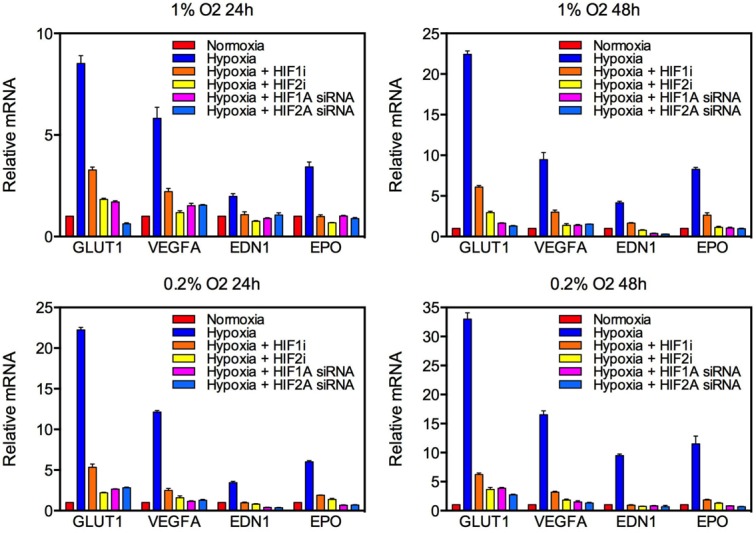
Knockdown of HIF targets by siRNA and pharmacologic agents in U87 glioblastoma cells Quantitative real-time PCR (qRT-PCR) was used to quantify mRNA expression of the HIF targets GLUT1, VEGFA, EDN1 and EPO following exposure to various oxygen concentrations for 24 (left) or 48 (right) hours. The *y-*axis reflects relative mRNA expression (standard errors of the means [S.E.M.]).

Given that antiangiogenic therapy can be associated with induction of a hypoxic phenotype and development of gliosarcoma, we hypothesized that exposure to hypoxia would enhance proliferation and mesenchymal change in GBM cell lines. U87 and U251 human GBM cells and C6 rat glioma cells were cultured in 21%, 1% or 0.2% oxygen for 24 or 48 hours. Expression of the HIF target genes coding for erythropoietin (EPO), vascular endothelial growth factor A (VEGFA), endothelin 1 (EDN1) and glucose transporter 1 (GLUT1) were markedly increased in both a time- and oxygen concentration-dependent manner, demonstrating robust activation of hypoxia signaling (Fig. 2, Fig. S1). Alternatively, expression of HIF targets was reduced by knockdown of HIF1α or HIF2α using small interfering RNA (siRNA) or pharmacologic inhibition (demonstrative of a robust blockade of HIF transcriptional activity).

**Figure 3:**
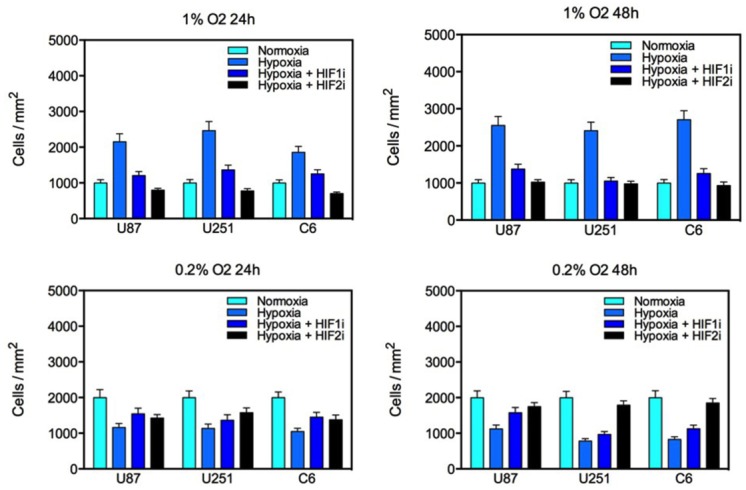
Proliferation of U87, U251 and C6 glioma cell lines under varying oxygen concentrations for 24 or 48 hours Culture in 1% oxygen saturation significantly increased glioma proliferation which was reduced by treatment with a HIF1α or HIF2α inhibitor. Culture in 0.2% oxygen saturation significantly reduced the number of viable cells in culture, and this effect was partially reversed by treatment with a HIF inhibitor. The *y*-axis represents the number of cells per square millimeter normalized to controls (S.E.M.).

To assess the effect of hypoxia on glioma cell proliferation, cell proliferation assays on glioma cells exposed to various oxygen concentrations for 24 or 48 hours were performed (Fig. 3). Under 1% oxygen tension, hypoxia reliably doubled the proliferative capacity of all glioma cell lines tested. This effect was reduced by pharmacological inhibition of HIF1α and HIF2α. Notably, inhibition of HIF2α more potently inhibited cell proliferation at 24 hours, but this effect was diminished by 48 hours. However, incubation at a very low oxygen concentration had the opposite effect with cell proliferation decreasing by roughly 50% at both time points. Consistent with a regulatory role for HIFs in GBM cell proliferation under hypoxic conditions, treatment with a HIF inhibitor partially rescued cell proliferation. Glioma cells exposed to 0.2% oxygen for 48 hours and incubated with the HIF2α inhibitor demonstrated very similar proliferative capacity to cells incubated under normoxic conditions. Consequently, the effect of hypoxia on glioma cell proliferation is both time- and concentration-dependent.

### Hypoxia induces mesenchymal change in glioma cells

**Figure 4:**
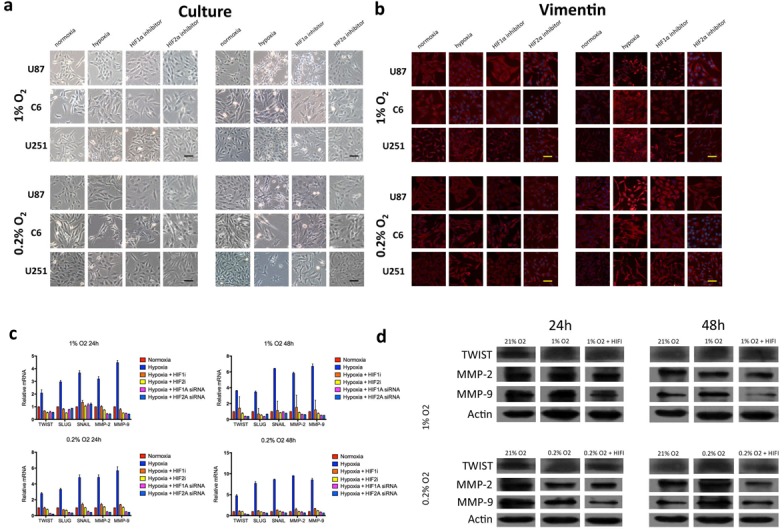
Morphologic changes and expression of EMT inducers and markers in glioma cell lines in response to hypoxia (a) Phase-contrast microscopy of U87, C6 and U251 glioma cell lines in culture under varying oxygen concentrations and treatment with a pharmacological inhibitor of HIF1α or HIF2α as indicated. (b) Immunofluorescent staining for the mesenchymal marker vimentin in each of the cultures from (a). Vimentin expression is upregulated in a time- and oxygen concentration-dependent manner and is significantly reduced by cell treatment with an inhibitor of HIF1α or HIF2α. (c) Quantitative real-time PCR of multiple EMT inducers, MMP2 and MMP9 extracted from U87 glioblastoma cells under various oxygen concentrations for 24 or 48 hours. Hypoxia consistently increased expression of EMT-associated genes, and this effect was blocked by treatment with HIFα inhibitors. The *y-*axis reflects relative mRNA expression (standard deviation [S.D.]). (d) Immunoblot of Twist, MMP2 and MMP9 extracted from U87 cells under the same conditions as (c). Hypoxia increased expression of each marker at 48 but not 24 hours, and the increase at 48 hours was significantly reduced by treatment with a HIF inhibitor. β-actin was used as a loading control.

To test the hypothesis that hypoxia induces mesenchymal change in glioblastoma, we evaluated the expression of the mesenchymal marker vimentin using immunofluorescence under various oxygen concentrations at 24 or 48 hours (Fig. 4a and 4b). Low levels of vimentin were detected under normoxic conditions in each of the cell lines evaluated, indicating some degree of mesenchymal change at baseline. At 24 hours, there was no appreciable change in vimentin expression under 1% oxygen concentration, and a slight increase was detected under 0.2% oxygen in U251 cells only. However, hypoxia consistently upregulated vimentin at 48 hours, indicating an oxygen concentration- and time-dependent acceleration of mesenchymal change under hypoxic conditions. This effect was reduced by treatment with a HIF inhibitor. This effect did not appear to differ between HIF1α and HIF2α.

Mesenchymal transition is governed by a defined set of transcription factors including Slug, Snail and Twist that repress genes coding for cell adhesion proteins, increase expression of MMPs and increase cell motility. To assess the effect of hypoxia on the expression of these transcription factors, we measured mRNA levels of Slug, Snail, Twist, MMP2 and MMP9 under various oxygen concentrations for 24 or 48 hours (Fig. 4c and S2). Exposure to either 0.2% or 1% oxygen saturation consistently upregulated genes associated with EMT in a time- and concentration-dependent fashion, demonstrating robust activation of the EMT program under hypoxia. Upregulation of EMT inducers and MMPs was consistently reduced following siRNA-mediated knockdown of HIF1α or HIF2α and after treatment with a HIF1α or HIF2α inhibitor.

To determine the time course of EMT-associated protein expression under hypoxia, immunoblots against Twist, Slug, MMP2 and MMP9 were assessed (Fig. 4d and S3). Each investigated target was expressed at high levels at baseline, indicating EMT inducers are present in glioma cell lines under normal conditions. Despite transcriptional upregulation of each target at less than 24 hours of hypoxia, there was no consistent change in Twist, MMP2 or MMP9 expression at 24 hours. However, at 48 hours under hypoxia, each EMT-associated protein was upregulated, and expression was blocked by treatment with a HIF inhibitor. These findings indicate that hypoxia enhances the expression of proteins that induce EMT and expression of these proteins is subject to inhibition by pharmacologic blockade of HIFs.

### Hypoxia enhances the migratory capacity of glioma cells

**Figure 5:**
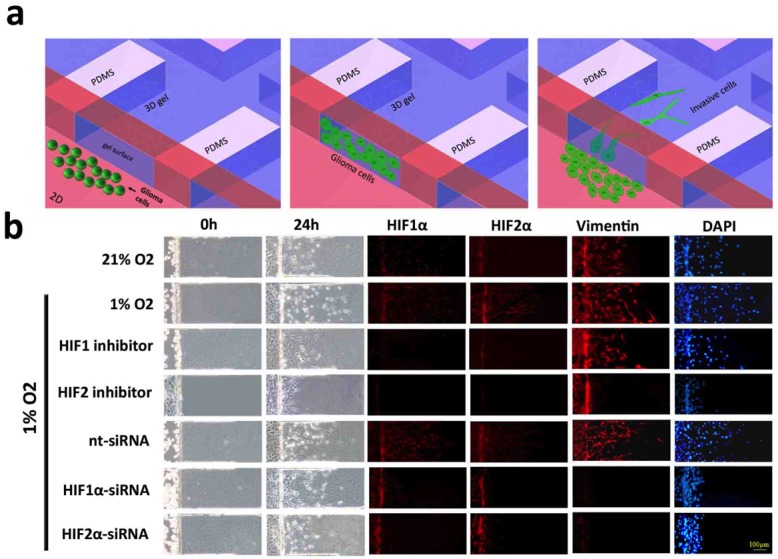
Microfluidic chip migration assay of U87 glioblastoma cells (a) Schematic diagram of the microfluidic chip migration assay. Cells are placed in a chamber containing either 21% or 1% oxygen and allowed to migrate along a three-dimensional collagen-based surface. (b) U87 glioma cell migration following exposure to 21% (top row) or 1% oxygen saturation for 24 hours. Immunofluorescent stains against HIF1α, HIF2 or vimentin are shown in red and DAPI is blue. Cells in the bottom five rows were treated with a HIF1α inhibitor, HIF2α inhibitor, non-targeting siRNA (nt-siRNA), HIF1α-siRNA or HIF2α-siRNA, as indicated. Hypoxia increased the distance and number of vimentin-positive cells that migrated onto the collagen surface. Total migration was significantly reduced after treatment with a HIF inhibitor and attenuated following HIF knockdown by siRNA. Treatment with a HIF2 inhibitor or siRNA knockdown of HIF1α or HIF2α significantly reduced the number of migrating vimentin-positive cells. Scale bar = 100 μm.

Mesenchymal transformation confers cells with enhanced migratory ability and is consistent with findings of diffuse spread of non-enhancing GBM on magnetic resonance (MR) imaging. To investigate whether hypoxia-induced mesenchymal transformation of glioma cells results in a migratory phenotype, we evaluated U87 GBM cell migration using a microfluidic chip invasion assay under 21% or 1% oxygen saturation (Fig. 5a). For this assay, cells were plated on a two-dimensional main channel, and invasive cells were allowed to migrate into a three-dimensional collagen matrix that more accurately simulated the microenvironment of the cell. Immunofluorescence was then performed to determine the expression of HIF1α, HIF2α and vimentin in migrated cells. Hypoxia significantly enhanced tumor cell migration, and invasive cells exhibited stronger vimentin expression than stationary cells (Fig. 5b). Although siRNA knockdown of HIF1/2α reduced glioma cell migration to levels seen under normoxia, the remaining migratory cells did not express the mesenchymal marker vimentin. Pharmacologic HIF blockade reduced cell migration even further, and this effect was more pronounced following HIF2α inhibition.

**Figure 6:**
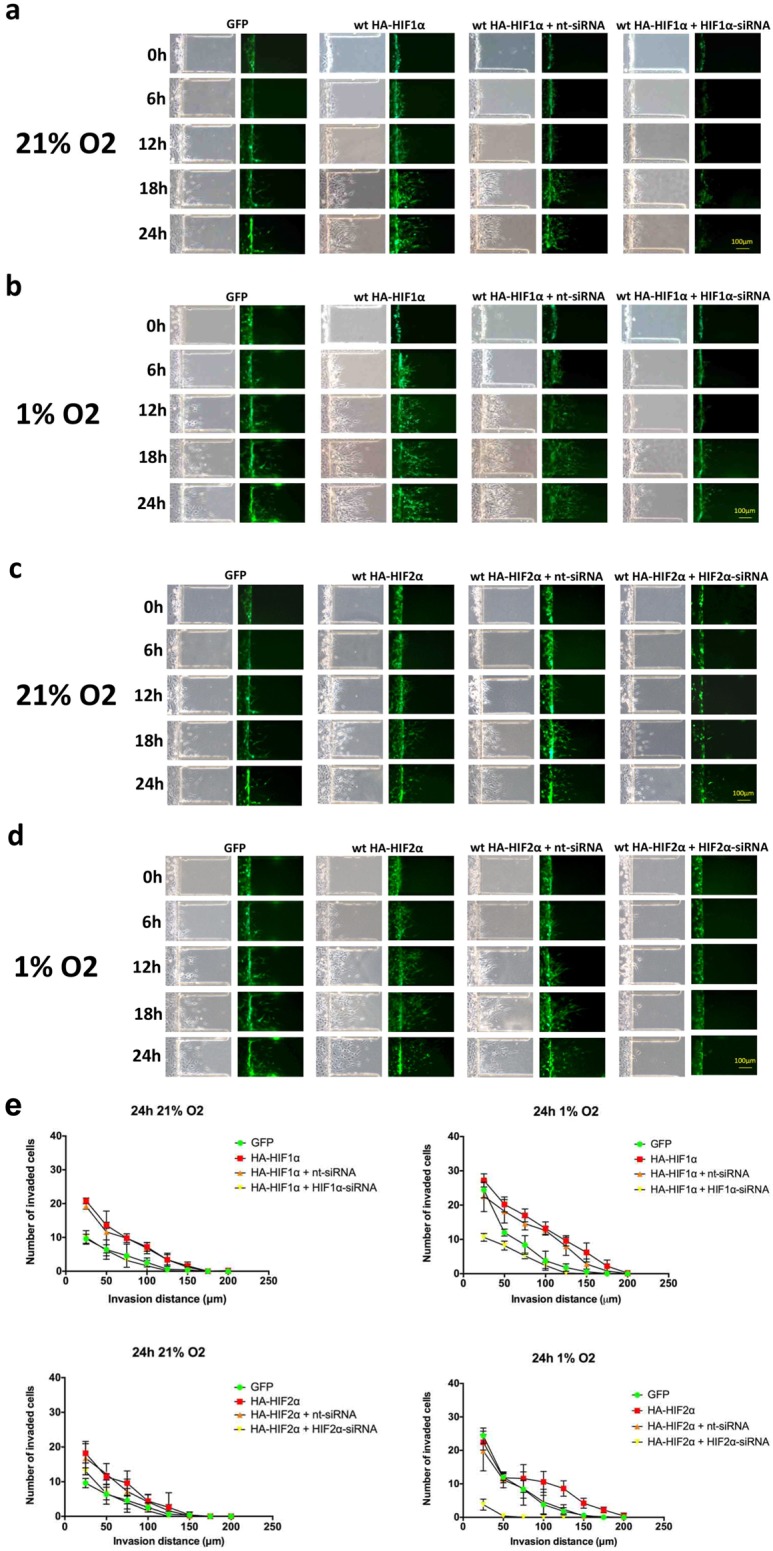
Time-course microfluidic chip migration assay of U87 glioma cells overexpressing HIF1α and HIF2α U87 glioma cells were transfected with GFP alone or GFP with a wild-type hemagglutinin-tagged (HA)-HIF1α gain-of-function construct. HIF1α gain-of-function cells were either transfected with HA-HIF1α alone or with nt-siRNA or HIF1α-siRNA. Cell cultures were then placed in the microfluidic chip assay, exposed to 21% (a) or 1% (b) oxygen saturation and imaged at 0, 6, 12, 18 and 24 hours, as indicated. The same set of experiments were performed using HA-HIF2α and HIF2α-siRNA in (c) and (d). Exposure to either hypoxia or transfection with HIF1α and HIF2α gain-of-function constructs enhanced cell migration, and migration was significantly reduced by siRNA knockdown of HIF1α and HIF2α, respectively. Scale bar = 100 μm. (e) Quantification of microfluidic chamber invasion assays shown in panels a-d. The *y-*axis reflects the number of invaded cells at each distance at 24 h (standard deviation [S.D.]).

Hypoxia is associated with changes in gene expression and metabolism that collectively contribute to its effect on cell phenotype. To determine the relative contribution of HIF1α and HIF2α to glioma cell migration, U87 GBM cells were transfected with hemagglutinin-tagged HIF α-subunit constructs with or without siRNA to a HIF α-subunit and then placed under 1% or 21% oxygen saturation for 24 hours (Fig. 6). Under normoxic conditions, cells that overexpressed HIFα migrated further than cells transfected with green fluorescent protein (GFP) alone. There was no discernible difference between HIF1α- and HIF2α-over-expressing cells. Migration was reduced following HIF1α knockdown and was nearly abolished following HIF2α knockdown. These findings indicate that both HIF1α and HIF2α are individually sufficient to enhance GBM cell migration and that HIF2α, in particular, is necessary for hypoxia-induced cell migration.

**Figure 7:**
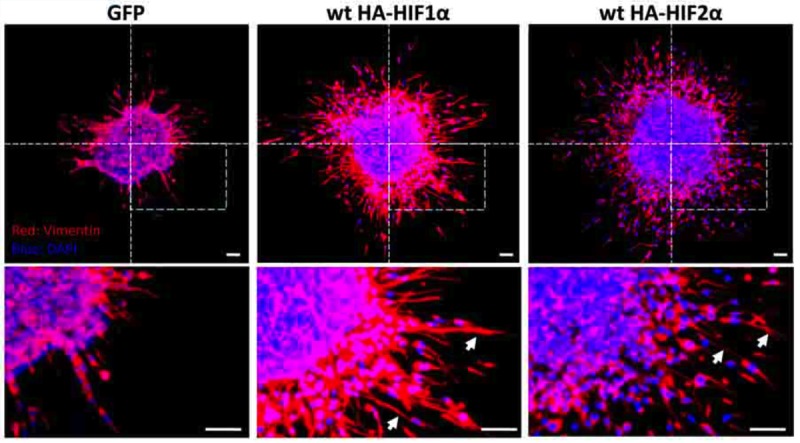
Neurosphere culture of U87 glioma cells under 1% or 21% oxygen Immunofluorescent stain for vimentin in neurosphere cultures. Vimentin-positive spindle-shaped cells (arrows) are visible migrating away from the neurosphere when HIF1α or HIF2α is over-expressed under normoxia. DAPI is blue. Scale bar = 100 μm.

To determine whether HIF overexpression is sufficient to induce migration in GBM stem cell populations, we performed a migration assay on neurospheres derived from U87 glioblastoma cells under 21% or 1% oxygen that selectively overexpressed HIF1α or HIF2α and then determined vimentin expression by immunofluorescence (Fig. 7). Overexpression of either HIF α-subunit was sufficient to enhance migration away from the neurosphere, indicating that HIFs are sufficient to stimulate migration out of GBM stem-cell populations.

## DISCUSSION

Hypoxia is a characteristic event in the progression of GBM. Rapidly proliferating tumor cells outstrip their blood supply leading to intratumoral necrosis and induction of the hypoxia signaling pathways. Bevacizumab is an anti-angiogenic therapeutic strategy that can further exacerbate hypoxia in solid tumors. Clinical data indicate that bevacizumab treatment can result in reduced tumor permeability (reduced enhancement on MR imaging) through VEGF-mediated mechanisms [23]. The reduction in vessel permeability results in reduced peritumoral edema and symptom improvement [24, 25]. Emerging data indicate that bevacizumab therapy is associated with non-enhancing (MR imaging) tumor progression and increased risk of gliosarcoma differentiation [10]. The results of the current study demonstrate that hypoxia activates mesenchymal transition and enhances cell motility in GBM in a HIF-dependent manner, and this process can be attenuated by pharmacological blockade of HIFα. Moreover, these data suggest that while HIF1α may preferentially enhance cell migration, HIF2α is necessary for this event to occur.

The term “epithelial-mesenchymal transition” was originally used to describe the process by which a cell adopts a fibroblast-like morphology and migratory phenotype in the process of normal embryonic development [26, 27]. Many of these features are recapitulated in cancer, which led to the observation that EMT is a critical process to cancer progression [26, 27]. Mesenchymal transition (and its converse, mesenchymal-epithelial transition) is now a well-described event in epithelial tumors and is increasingly recognized as a key event in non-epithelial cancers such as GBM [26-34]. Mesenchymal transition confers multiple oncogenic properties to cancer cells. In addition to enhancing their invasive capacity, EMT endows differentiated cancer cells with the capacity to become stem cells and provides a mechanism for their continued production [26, 27, 34, 35]. EMT also plays a key role in chemoresistance: Zeb1, for example, controls the susceptibility of GBM to temozolomide by disinhibiting the transcription factor c-MYB, a transcriptional activator of the DNA repair enzyme O-6-Methylguanine DNA Methyltransferase (MGMT) [32].

In the context of new therapeutic approaches to GBM, mesenchymal transition is a key event in resistance to anti-angiogenic therapy [10, 36]. Recent studies have demonstrated that VEGF antagonists, although providing a temporary reduction in enhancing tumor volume and peritumoral edema, ultimately fail and can induce a mesenchymal phenotype that is more invasive and resistant to therapy than the original tumor [10]. Mechanisms of resistance to VEGF antagonists are multiple. VEGF blockade disinhibits the HGF receptor c-MET, resulting in constitutive activation of tyrosine kinase-ras signaling in GBM [36]. VEGF-resistant cancer cells also secrete significantly more IL-17, resulting in the recruitment of immature immune cells to the tumor microenvironment [37]. VEGF inhibition directly induces tissue hypoxia by relative vasoconstriction, disinhibition of endothelial cell apoptosis and subsequent vessel collapse [38-40]. Intratumoral hypoxia has long been recognized as a sign of successful antiangiogenic therapy in solid tumors but ultimately limits its therapeutic potential by enhancing the proliferative, invasive and stem-like properties of tumor cells [13].

Hypoxia is a potent inducer of EMT. Hypoxia-inducible factors activate transcription of multiple EMT-related proteins that leads to a loss of cell-cell adhesion, increased cell motility and evasion from cell cycle arrest [41, 42]. This process is crucially dependent on HIF stabilization, as EMT in *VHL*-null renal cell carcinoma occurs in the absence of true hypoxia [41]. Under normoxic conditions, the E3 ubiquitin ligase von Hippel Lindau protein (pVHL) ubiquitinates hydroxylated HIF α-subunits, targeting them for degradation by the proteasome [43]. Under hypoxic conditions or conditions where HIF α-subunits are otherwise stabilized, the inducible HIFα-subunits translocate to the nucleus where they form transcriptionally active heterodimers with constitutively expressed HIF α-subunits [44]. Activation of the hypoxia signaling pathway is associated with increased activation of the EMT program, increased cell motility and adoption of stem-like traits and is thus an attractive therapeutic target for solid tumors [27, 35, 41, 42, 45-49].

Our data demonstrate that hypoxia is a potent enhancer of mesenchymal transition and cell motility in GBM. These events can be significantly attenuated by treatment with a pharmacologic inhibitor of HIFs. Given that anti-angiogenic therapy can be associated with an increase in intratumoral hypoxia, these findings provide a mechanism for the observed non-enhancing progression of tumor and the increase in frequency of secondary gliosarcoma transformation following bevacizumab therapy. These data offer the possibility that adjuvant therapy with a HIF inhibitor can delay GBM progression during anti-angiogenic therapy.

## MATERIALS AND METHODS

### Human tissue collection

Surgical specimens from patient 1 were obtained from the Department of Neurosurgery at Huashan Hospital of Fudan University, Shanghai, China. Informed consent was obtained according to Institutional Review Board at Huashan Hospital. Brain autopsies of patients 2 and 3 were performed at The Ohio State University and tissue was obtained through The Ohio State University Department of Pathology. Informed consent was obtained from both patients and approved by the Institutional Review Board of the Office of Responsible Research Practices at The Ohio State University Wexner Medical Center. All specimens were subjected to fixation in 10% formalin and subsequent paraffin blocking. 5 μm-thick sections were obtained from each paraffin block and utilized for hematoxylin and eosin (H&E) and immunohistochemical staining.

### HIF knockdown and gain-of-function conditions

Small-interfering RNA and oligonucleotides against human HIF-1α, human HIF-2α, rat HIF-1α and rat HIF-2α and hemagglutinin-tagged gain-of-function constructs were purchased from Sigma-Aldrich (St. Louis, MO). Sequences are listed in Table S1. Cells were transfected with siRNA at a concentration of 75nM using Lipofectamine 2000 Transfection Reagent (Life Technologies, Carlsbad, CA). Pharmacologic inhibition of HIFs was achieved using an inhibitor of HIF1α-mediated transcription (methyl-3-[[2-[4-(2-adamantyl)phenoxy]acetyl]amino]-4-hydroxybenzoate) (Santa Cruz Biotechnology, Santa Cruz, CA) or HIF2α translation (Methyl-3-(2-(cyano(methylsulfonyl)methylene)hydrazino)thiophene-2-carboxylate) (Merck Millipore, Darmstadt, Germany) at a concentration of 30μM in DMSO.

### Microfluidic chip construction

The microfluidic chip consisted of a bilayer of polydimethylsiloxane (PDMS) and glass substrate. All chips were designed using an SU8-2035 negative photoresistor (Micro Chem Corp., Newton, MA) molded from a master PDMS layer. Negative chip molds were spun onto a glass wafer and patterned photolithographically in duplicate (100 mm thickness for lower layer and 190 μm thickness for upper layer), producing unique microstructures with different heights. PDMS base and curing agent (Sylgard Silicone elastomer 184, Dow Corning Corp., Washington, D.C.) were mixed thoroughly 10:1 by mass, degassed under vacuum and poured onto the master. The mold curing process was conducted for 1 h at 80°C. After cooling, the PDMS layer was gently peeled off of the master and trimmed to size. Inlet and outlet holes were created by punching through the PDMS with a razor-sharp punch. The PDMS mold was decontaminated with oxygen plasma for 15 s, bonded to a glass slide and sterilized with UV light for 30 m. Rat Tail High-Concentration Type-I Collagen solution (BD Biosciences, Franklin Lakes, NJ) was compounded according to an alternate gelation procedure, aseptically added into collagen chamber of the chip and allowed to gel at 37°C for 30 m.

### Microfluidic chip invasion assay

Cells were seeded into the main channel of the microfluidic chip at a density of 5 × 10^4^ cells/cm^2^ with medium containing 10% FBS. The chip was then turned on its side for 5 m to allow the cells to adhere to the surface of the gel. Each chip was then incubated for either 24 h at 37°C, 21% O_2_, 5% CO_2_ in a humidified incubator (normoxic condition) or at 37°C in an electric microscope stage incubation chamber (Okolab, Ottaviano, Italy) flushed with a gas mixture of 1% O_2_, 5% CO_2_, 94% N_2_ or 0.2% O2, 5% CO2, 94% N2 (hypoxic conditions). Cells were allowed to invade for 24 h, and images were recorded every 6 h by an Olympus IX71 fluorescence microscope equipped with a CCD digital camera (Olympus, Tokyo, Japan). After 24 h, each chip was stained with primary antibody and prepared for immunofluorescent imaging as described above.

## SUPPLEMENTARY MATERIAL FIGURES AND TABLES


