# Supplementary material for: Activation of hypoxia signaling induces phenotypic transformation of glioma cells: implications for bevacizumab antiangiogenic therapy
Source: Oncotarget. 2015 Mar 14;6(14):11882–93. doi: 10.18632/oncotarget.3592 (PMC4494911; doi:10.18632/oncotarget.3592)
Supplement: Supplementary file 1 [file oncotarget-06-11882-s001.pdf]

Activation of hypoxia signaling induces phenotypic transformation of glioma cells: implications for bevacizumab antiangiogenic therapy

Supplementary Material

MATERIALS AND METHODS

Immunohistochemistry

Paraffin-embedded tissue samples were de-paraffinized with xylene and dehydrated with ethanol. Epitope retrieval was performed using a 1% citrate solution and Decloaker (Nobel Biocare, Zurich, Switzerland). Cells were permeabilized using 0.5% hydrogen peroxide in methanol. Slides were blocked in 1% TBSA and Background Punisher (Nobel Biocare) and incubated with primary antibody overnight. Primary antibody was visualized using MACH4 horseradish peroxidase (Nobel Biocare) and DAB (Vector Laboratories, Burlingame, CA). Slides were subsequently dipped in Hematoxylin-Mayers solution, dehydrated in ethanol, wet with xylene and coverslipped with Permount (Fisher Scientific, Waltham, MA). The primary antibodies used for immunohistochemistry included rabbit polyclonal HIF1α (Novus Biologics, Littleton, CO), rabbit polyclonal anti-Zeb1 (Sigma-Aldrich, St. Louis, MO), rabbit polyclonal anti-Zeb2 (Sigma-Aldrich), rabbit polyclonal anti-Slug/Snail (Abcam, Cambridge, MA), rabbit polyclonal anti-Twist (Abcam) and mouse monoclonal anti-MMP-2 (Millipore, Billerica, MA). Images were acquired using a Nikon (Tokyo, Japan) ECLIPSE Ci-E light microscope coupled to a Nikon DS Fi2 camera.

Cell culture

Human U87, human U251 and rat C6 glioma cell lines were obtained from the Cell Bank of the Chinese Academy of Sciences (Shanghai, China) and cultured according

to their respective protocols as recommended by the manufacturer. Briefly, cells were cultured in Dulbecco's Modified Eagle Medium (DMEM) basic (Life Technologies, Carlsbad, CA) in 10% FBS at 37°C, 21% O_2, 5% CO_2 in a humidified incubator (normoxic condition) or at 37°C in an electric microscope stage incubation chamber (Okolab, Ottaviano, Italy) flushed with a gas mixture of 1% O_2, 5% CO_2, 94% N_2 or 0.2% O_2, 5% CO_2, 94% N_2 (hypoxic conditions). Culture medium was replaced after rinsing cells with D-PBS Phenol Red every two days. Cells were digested using 1X 0.25% Trypsin-EDTA (Life Technologies) for experiments.

RNA isolation and quantitative real-time PCR

Total RNA was isolated from cells using RNAiso Plus (Takara, Shiga, Japan). The concentration of total RNA was determined by measuring absorbance at 260nm and 280 nm after diluting the RNA solution with DEPC-treated water and calculated as the ratio of OD260/OD280. RNAs with the OD260/OD280 ratio between 1.7 and 2.1 were used for quantification. cDNA was synthesized using a PrimeScript™ RT reagent Kit (Perfect Real Time, Takara) according to the manufacturer's protocol for the SYBR Green assay. Real-time PCR was carried out using SYBR® Premix Ex Taq™ II Kit (Tli RNaseH Plus, Takara) in a Mx3000P QPCR system (Agilent Technologies, Santa Clara, CA). Real-time PCR conditions were as follows: 1 cycle, 95°C, 30s; 40 cycles, 95°C, 5s, 55°C, 30s, 72°C, 30s; 1 cycle, 95°C, 15s, 60°C, 1min, 95°C, 15s. Melting curve analysis was performed to confirm the authenticity of the PCR product. The RT-PCR primers were synthesized by Takara and sequences are listed in Supplementary Table 2. *ACTB* expression was used as normalize mRNA expression, and relative quantitation of mRNA

expression levels were determined and calculated by MxPro QPCR software (Agilent Technologies).

Morphologic evaluation of cells

Cells were seeded at a density of 5×10^5 cells/mL and subject to either the normoxic or hypoxic condition as described above. Cell morphology was evaluated by phase-contrasted microscopy using an Olympus IX71 microscope equipped with a CCD digital camera (Olympus Corporation, Tokyo, Japan).

Immunofluorescence and morphologic evaluation of cells

Cells were fixed in 4% paraformaldehyde and permeabilized with 0.1% Triton X-100 solution. Samples were then blocked in 1% TBSA and incubated with primary antibody diluted in PBS overnight. Primary antibody was detected by incubating cells in fluoropore-conjugated secondary antibodies diluted in PBS for 1h away from light, and nuclei were counterstained with DAPI (Sigma-Aldrich, St. Louis, MO). Images were acquired using an Olympus IX71 fluorescence microscope equipped with CCD digital camera (Olympus Corporation) and processed using Image-Pro Plus software (Media Cybernetics, Rockville, MD). Primary antibodies used for immunofluorescence included rabbit polyclonal anti-HIF1α (Proteintech, Chicago, IL), mouse monoclonal anti-HIF2α (R&D Systems, Minneapolis, MN) and mouse monoclonal anti-vimentin (Beijing Zhongshan Golden Bridge Biological Technology Co., Ltd., Beijing, China) and were diluted according to manufacturer recommendations. Fluoropore-conjugated secondary antibodies included Rhodamine (TRITC)-conjugated AffiniPure Goat Anti-Mouse IgG (H+L) and Fluorescein (FITC)- Conjugated AffiniPure Goat Anti-Rabbit IgG (H+L)

(Beijing Zhongshan Golden Bridge Biological Technology Co., Ltd.) and were diluted to a concentration of 1:100.

Cell proliferation assay

Cell proliferation was assessed by counting the number of viable cells in four randomly chosen high power fields. For each experiment, the average number of cells was adjusted for the seeding density of the control condition. Cells were plated on a 6-well plate (Corning Incorporated, Corning, NY) at a density of 1×10^3 cells per mm^2 or 2×10^3 cell per mm^2 and incubated in 21% O_2, 1% O_2 or 0.2% O_2 as described above.

Immunoblot

All protein samples were separated with 10 % sodium dodecyl sulfate gel electrophoresis (SDS-PAGE). Following transfer, membranes were blocked in 5 % skim milk in tris-buffered saline with TWEEN-20 (TBST, 10mM Tris-HCl, pH 7.6; 150 mM NaCl; 0.5% TWEEN-20) for 2 h at room temperature. Blocked membranes were incubated with primary antibody diluted in PBS overnight at 4 °C. Membranes were then washed with TBST and incubated with HRP-conjugated secondary antibody for 2 h at room temperature and developed with an enhanced electrochemiluminescence (ECL) Plus procedure as specified by the manufacturer (Beyotime Institute of Biotechnology; Shanghai, China). Relative protein density was analyzed with a BioSpectrum-410 multispectral imaging system with a Chemi HR camera 410 (UVP, USA). β-actin was used as an internal control. Primary antibodies used for immunoblot included anti-β-actin (1:2000), rabbit polyclonal anti-MMP-2 (Bioworld Technology, Minneapolis, MN), rabbit polyclonal anti-MMP-9 (Bioworld Technology), rabbit monoclonal anti-Slug (Cell

Signaling Technology, Danvers, MA) and mouse monoclonal anti-Twist (Sigma-Aldrich) and were diluted to 1:200 concentration.

Neurosphere culture

Cells were transfected with GFP, HA-HIF1α or HA-HIF2α and placed in a cell culture dish (Corning) at a density of 2.5×10^5 cells/mL. Each dish was then inverted and placed in a humidified incubator for 48h for spheroid generation. Spontaneously formed spheroids were centrifuged and harvested. Type-I collagen solution (BD Biosciences) was diluted to a working concentration of 3mg/mL in a well plate for resuspension and dispersion of the collected spheroids. The collagen solution was allowed to sit at 37°C for 30 m for cross-linking and solidification. Spheroids and culture medium was then added to each collagen block and incubated in a humidified incubator at 37°C for 24 h. Tumor spheroids were then fixed in the collagen matrix and subject to immunofluorescence as described above. The number of cells that invaded the collagen gel and the distance migrated were quantified using Image-Pro Plus software (Media Cybernetics, Rockville, MD).

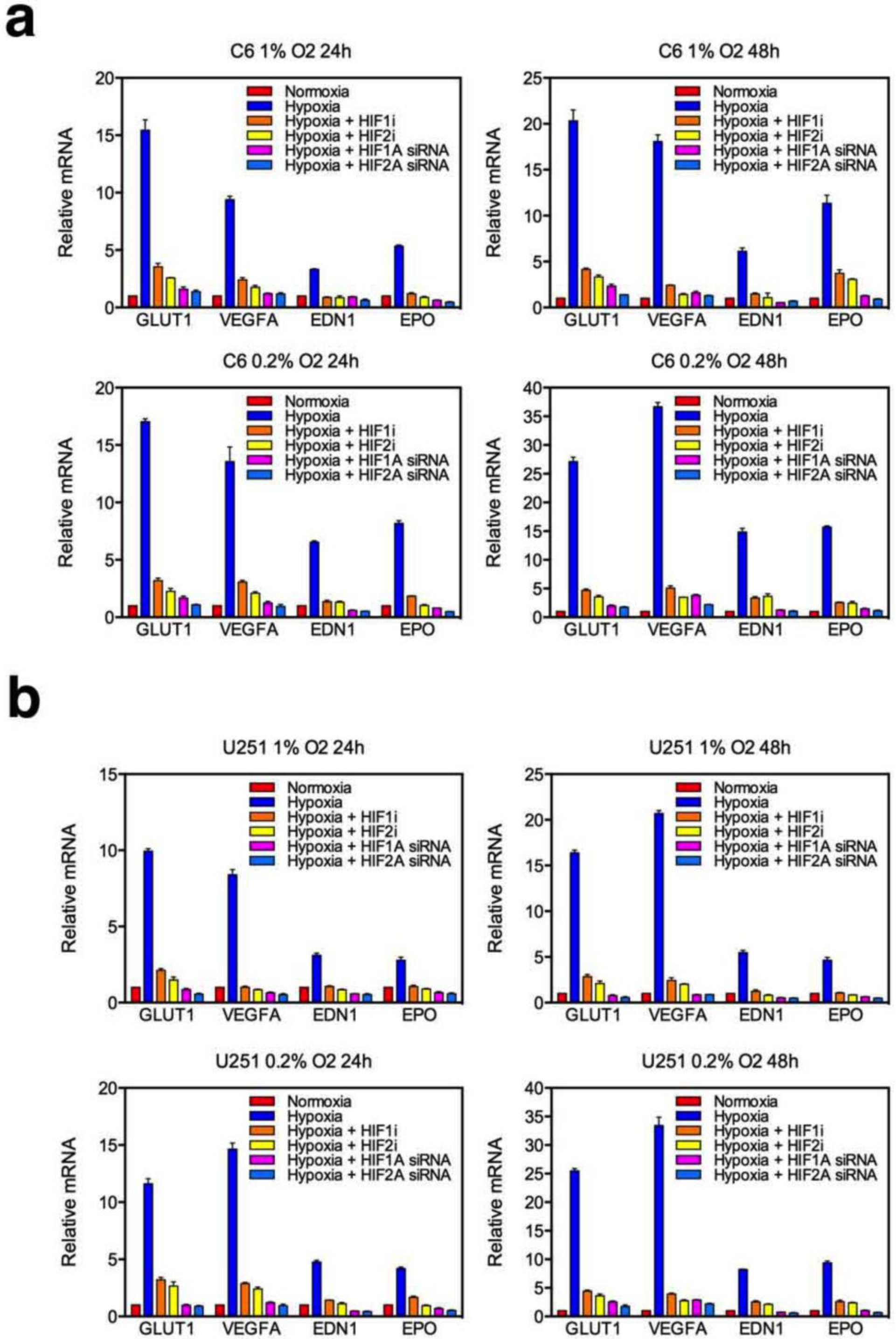

Figure S1: Knockdown of HIF targets by siRNA and pharmacologic agents. Relative mRNA levels are shown in C6 **(a)** and U251 **(b)** glioma cell lines. Quantitative real-time

PCR (qRT-PCR) was used to quantify mRNA expression of the HIF targets GLUT1, VEGFA, EDN1 and EPO following exposure to various oxygen concentrations for for 24 (left) or 48 (right) hours. The *y*-axis reflects relative mRNA expression (standard errors of the means [s.e.m]).

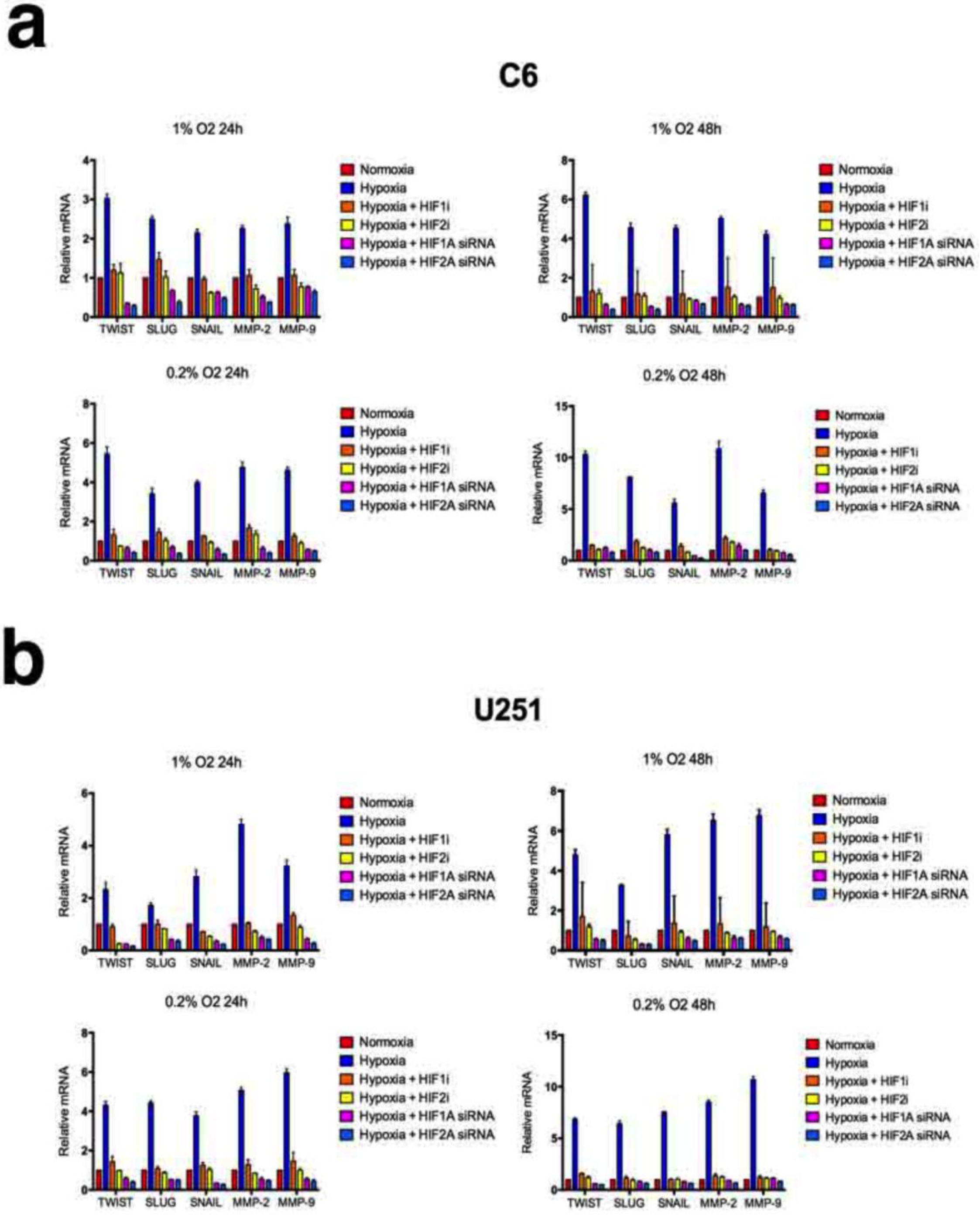

Figure S2. Quantitative real-time PCR of multiple EMT inducers, MMP2 and MMP9. Relative mRNA levels are shown from C6 **(a)** and U251 **(b)** glioma cells under various oxygen concentrations for 24 or 48 hours. Hypoxia increased expression of EMT-

associated genes, and this effect was blocked by treatment with a HIF inhibitor. The *y*-axis reflects relative mRNA expression (standard deviation [S.D.]).

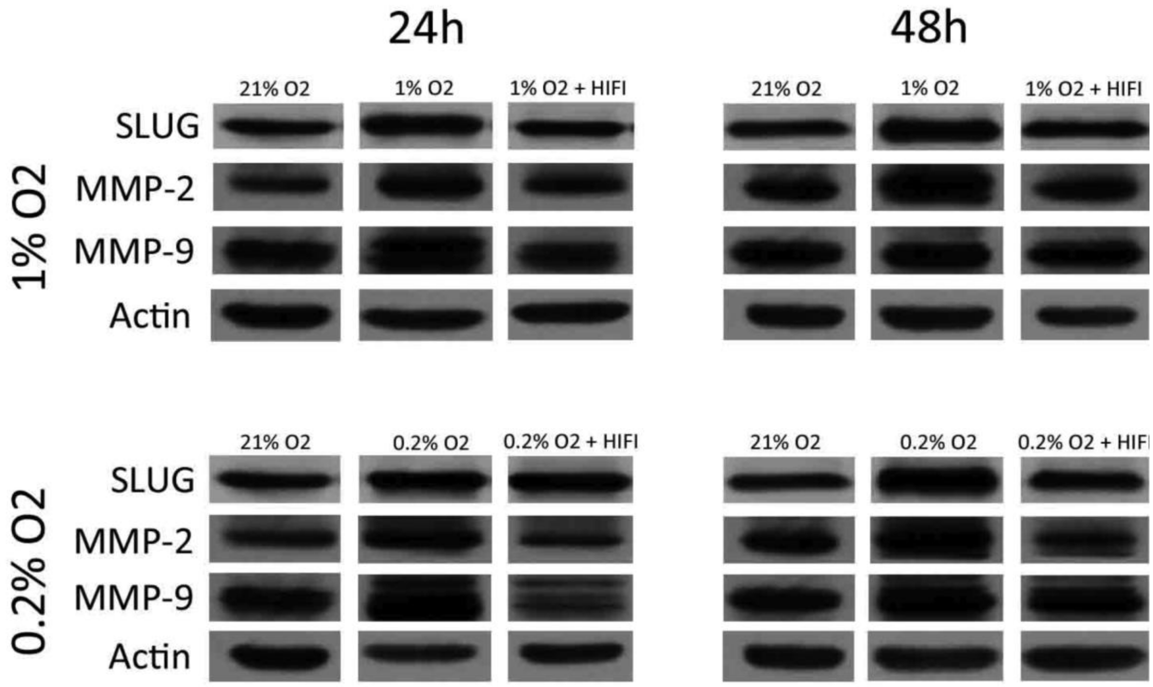

Figure S3. Immunoblot of Slug, MMP2 and MMP9 extracted from U251 glioblastoma cells under various oxygen concentrations for 24 or 48 h. Hypoxia increased expression of each marker, and EMT marker expression was significantly reduced by treatment with a HIF inhibitor. β-actin was used as a loading control.

Table S1. Small-interfering RNAs.

Target gene	siRNA Start	siRNA sequences	Accession number
HIF-1A (Human)	1801	sense strand: 5'- CAAAGUUCACCUGAGCCUAdTdT -3'<br>antisense strand: 5'- UAGGCUCAGGUGAACUUUGdTdT -3'	NM_001530.3
HIF-1A (Human)	642	sense strand: 5'- GAUUAACUCAGUUUGAACUdTdT -3'<br>antisense strand: 5'- AGUUCAAACUGAGUUAAUCdTdT -3'	NM_001530.3
HIF-1A (Human)	1549	sense strand: 5'- GCAACUUGAGGAAGUACCAdTdT -3'<br>antisense strand: 5'- UGGUACUUCCUCAAGUUGCdTdT -3'	NM_001530.3
HIF-2A (Human)	2750	sense strand: 5'- GCAAAUGUACCCAAUGAUAdTdT -3'<br>antisense strand: 5'- UAUCAUUGGGUACAUUUGCdTdT -3'	NM_001430.4
HIF-2A (Human)	911	sense strand: 5'- GAGAUUCGUGAGAACCUGAdTdT -3'<br>antisense strand: 5'- UCAGGUUCUCACGAAUCUCdTdT -3'	NM_001430.4
HIF-2A (Human)	2301	sense strand: 5'- CAUCUUCUUUGAUGCCGGAdTdT -3'<br>antisense strand: 5'- UCCGGCAUCAAAGAAGAUGdTdT -3'	NM_001430.4
HIF-1A (Rat)	2028	sense strand: 5'- GGAAAGAGAGUCAUAGAAAdTdT -3'<br>antisense strand: 5'- UUUCUAUGACUCUCUUUCCdTdT -3'	NM_024359.1
HIF-1A (Rat)	2211	sense strand: 5'- GAAUUGGAACGUUACUGCAdTdT -3'<br>antisense strand: 5'- UGCAGUAACGUUCCAAUUCdTdT -3'	NM_024359.1
HIF-1A (Rat)	1108	sense strand: 5'- CAGUUGAAUCUUCAGAUAUdTdT -3'<br>antisense strand: 5'- AUAUCUGAAGAUUCAACUGdTdT -3'	NM_024359.1
HIF-2A (Rat)	433	sense strand: 5'- GACUUACCCAGGUAGAACUdTdT -3'<br>antisense strand: 5'- AGUUCUACCUGGGUAAGUCdTdT -3'	NM_023090.1
HIF-2A (Rat)	1550	sense strand: 5'- CACUUGAAGAUCGAAGUAAdTdT -3'<br>antisense strand: 5'- UUACUUCCAUCUUCAAGUGdTdT -3'	NM_023090.1
HIF-2A (Rat)	1893	sense strand: 5'- CUUUGAUGCUGGGAGCAAAdTdT -3'<br>antisense strand: 5'- UUUGCUCCCAGCAUGAAAGdTdT -3'	NM_023090.1

Table S2: Quantitative real-time PCR primer sets.

Target gene	Primer sequences	Accession number
ACTB (Human)	forward: 5'- TGGCACCCAGCACAATGAA -3'<br>reverse: 5'- CTAAGTCATAGTCCGCCTAGAAGCA -3'	NM_001101.3
GLUT1 (Human)	forward: 5'- CGGGCCAAGAGTGTGCTAAA -3'<br>reverse: 5'- TGACGATACCGGAGCCAATG -3'	NM_006516.2
VEGFA (Human)	forward: 5'- GTGCCCGCTGCTGTCTAATG -3'<br>reverse: 5'- TCTGCGGATCTTGTACAAACAAATG -3'	NM_001025366.2
EDN1 (Human)	forward: 5'- TTATCAGCAGTTAGTGAGAGG -3'<br>reverse: 5'- GAAGGTCTGTCACCAATGTG -3'	NM_001955.4
EPO (Human)	forward: 5'- ACTGCAGCTTGAATGAGAATATCAC -3'<br>reverse: 5'- AGGACAGCTTCCGACAGCA -3'	NM_000799.2
TWIST1 (Human)	forward: 5'- GTCCGCAGTCTTACGAGGAG -3'<br>reverse: 5'- CCAGCTTGAGGGTCTGAATC -3'	NM_000474.3
SLUG (Human)	forward: 5'- CAAGGACACATTAGAACTCACAC -3'<br>reverse: 5'- CTACACAGCAGCCAGATTC -3'	NM_003068.4
SNAIL (Human)	forward: 5'- CATCCTTCTCACTGCCATGGA -3'<br>reverse: 5'- AGGCAGAGGACACAGAACCAGA -3'	NM_005985.3
MMP-2 (Human)	forward: 5'- CCGTCGCCCATCATCAAGTT -3'<br>reverse: 5'- CTGTCTGGGGCAGTCCAAAG -3'	NM_004530.4
MMP-9 (Human)	forward: 5'- GGGACGCAGACATCGTCATC -3'<br>reverse: 5'- TCGTCATCGTCGAAATGGGC -3'	NM_004994.2
ACTB (Rat)	forward: 5'- AGGGAAATCGTGCGTGAC -3'<br>reverse: 5'- CGCTCATTGCCGATAGTG -3'	NM_031144.3
GLUT1 (Rat)	forward: 5'- GACCCTGCATCTCATTGGTCTG -3'<br>reverse: 5'- CCACAATGAACCATGGAATAGGA -3'	NM_138827.1
VEGFA (Ra)	forward: 5'- GCACGTTGGCTCACTTCCAG -3'<br>reverse: 5'- TGGTCGGAACCAGAATCTTTATCTC -3'	NM_031836.2
EDN1 (Rat)	forward: 5'- ACCTGGACATCATCTGGGTCAAC -3'<br>reverse: 5'- TTTGGTGAGCACACTGGCATC -3'	NM_012548.2
EPO (Rat)	forward: 5'- TACGTAGCCTCACTTCACTGCTTC -3'<br>reverse: 5'- CCCGTGTACAGCTTCAGTTTCC -3'	NM_017001.1
TWIST1 (Rat)	forward: 5'- ACCCTCACACCTCTGCATTC -3'<br>reverse: 5'- CAGTTTGATCCCAGCGTTTT -3'	NM_053530.2

SLUG (Rat)	forward: 5'- GCACTGTGATGCCCAGGCTA -3'<br>reverse: 5'- CCTTGCCACAGATCTTGCAGAC -3'	NM_013035.1
SNAIL (Rat)	forward: 5'- ACCCACACTGGTGAGAAGCC -3'<br>reverse: 5'- TTCACATCCGAGTGGGTCTG -3'	NM_053805.1
MMP-2 (Rat)	forward: 5'- GCTGATACTGACACTGGTACTG -3'<br>reverse: 5'- CAATCTTTTCCGGGAGCTC -3'	NM_031054.2
MMP-9 (Rat)	forward: 5'- AAGGATGGTCTACTGGCAC -3'<br>reverse: 5'- AGAGATTCTCACTGGGGC -3'	NM_031055.1
